# Supplementary material for: The effect of intrapartum prolonged oxygen exposure on fetal metabolic status: secondary analysis from a randomized controlled trial
Source: Front Endocrinol (Lausanne). 2023 Jun 27;14:1204956. doi: 10.3389/fendo.2023.1204956 (PMC10335765; doi:10.3389/fendo.2023.1204956)
Supplement: Supplementary file 3 [file DataSheet_3.zip › Result-X101SC21092977-Z01-J001-B1-42/4.MetDiffAnalysis/O.vs.A/O.vs.A_pos_cluster_heatmap.pdf]

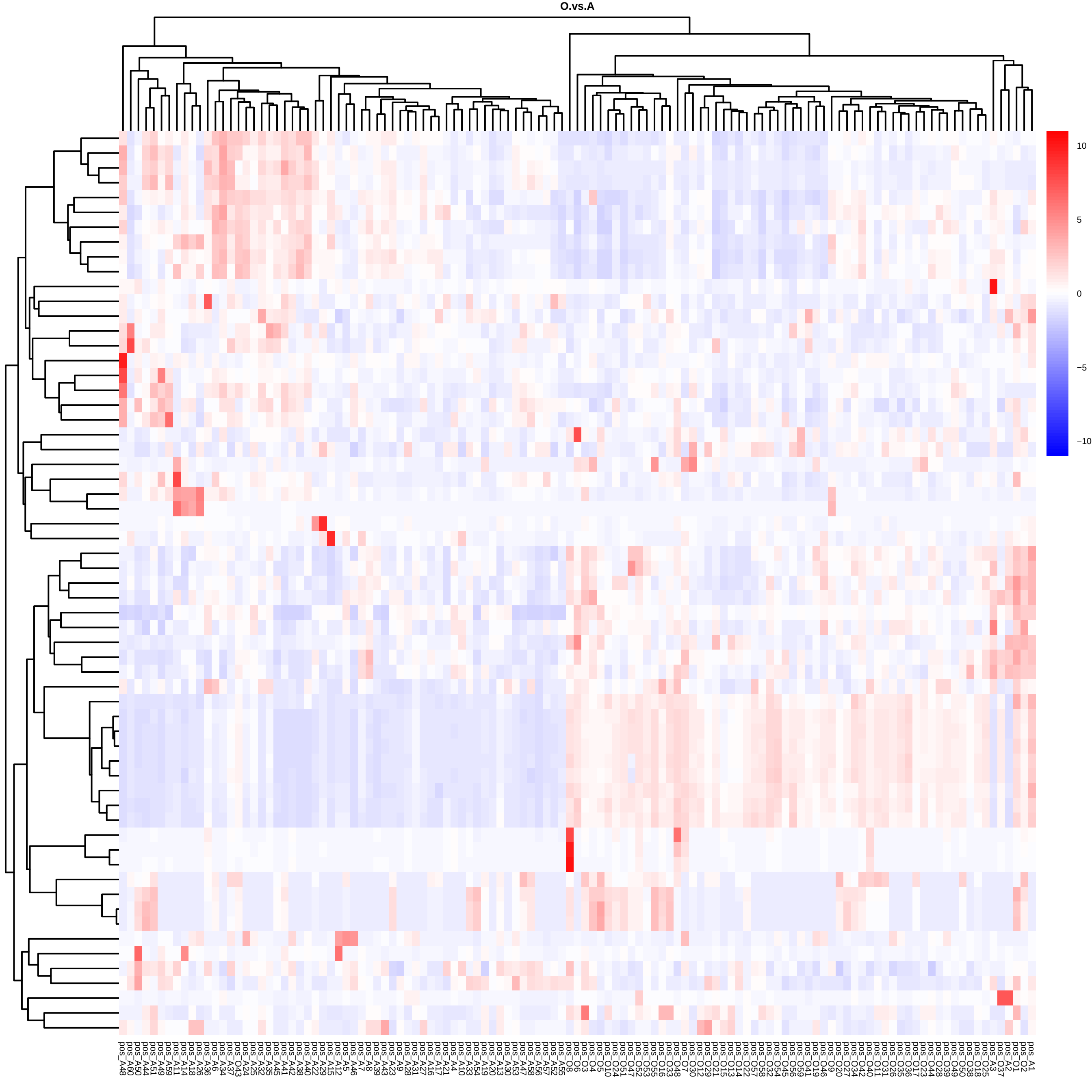
O.vs.A
10
5
0
−5
−10
pos_A48
pos_A60
pos_A50
pos_A44
pos_A51
pos_A49
pos_A59
pos_A11
pos_A14
pos_A18
pos_A26
pos_A36
pos_A6
pos_A34
pos_A37
pos_O43
pos_A24
pos_A25
pos_A32
pos_A35
pos_A45
pos_A41
pos_A42
pos_A38
pos_A40
pos_A22
pos_A29
pos_A15
pos_A12
pos_A5
pos_A46
pos_A7
pos_A8
pos_A39
pos_A43
pos_A23
pos_A9
pos_A28
pos_A31
pos_A27
pos_A16
pos_A17
pos_A21
pos_A4
pos_A10
pos_A33
pos_A54
pos_A19
pos_A20
pos_A13
pos_A30
pos_A53
pos_A47
pos_A58
pos_A56
pos_A57
pos_A52
pos_A55
pos_O8
pos_O6
pos_O3
pos_O4
pos_O5
pos_O10
pos_O24
pos_O51
pos_O47
pos_O52
pos_O53
pos_O55
pos_O16
pos_O33
pos_O48
pos_O7
pos_O30
pos_O12
pos_O29
pos_O21
pos_O15
pos_O13
pos_O14
pos_O22
pos_O57
pos_O58
pos_O32
pos_O54
pos_O45
pos_O56
pos_O59
pos_O41
pos_O19
pos_O46
pos_O9
pos_O20
pos_O27
pos_O34
pos_O42
pos_O40
pos_O11
pos_O31
pos_O26
pos_O35
pos_O36
pos_O17
pos_O23
pos_O44
pos_O28
pos_O39
pos_O49
pos_O50
pos_O38
pos_O18
pos_O25
pos_A3
pos_O37
pos_A2
pos_O1
pos_O2
pos_A1
